# *Bacillus licheniformis* and *Bacillus subtilis*, Probiotics That Induce the Formation of Macrophage Extracellular Traps

**DOI:** 10.3390/microorganisms9102027

**Published:** 2021-09-25

**Authors:** Carol M. Romo-Barrera, Laura E. Castrillón-Rivera, Alejandro Palma-Ramos, Jorge I. Castañeda-Sánchez, Julieta Luna-Herrera

**Affiliations:** 1 Departamento de Inmunología, Escuela Nacional de Ciencias Biológicas, Instituto Politécnico Nacional, Ciudad de México 11340, Mexico; camayro@gmail.com; 2 Departamento de Sistemas Biológicos, Universidad Autónoma Metropolitana Unidad Xochimilco, Ciudad de México 04960, Mexico; lcrivera@correo.xoc.uam.mx (L.E.C.-R.); alpalma@correo.xoc.uam.mx (A.P.-R.)

**Keywords:** probiotic, *Bacillus licheniformis*, *Bacillus subtilis*, macrophage extracellular trap, bacillus

## Abstract

Probiotics are considered living microorganisms that help preserve the health of the host who uses them. *Bacillus* are a genus of Gram-positive bacteria used as probiotics for animal and human consumption. They are currently distributed in various commercial forms. Two of the species used as probiotics are *B. licheniformis* and *B. subtilis*. Macrophages are central cells in the immune response, being fundamental in the elimination of microbial pathogens, for which they use various mechanisms, including the formation of extracellular traps (METs). There have been very few studies carried out on the participation of macrophages in response to the interaction of probiotics of the genus *Bacillus* with the host. In this work, we used macrophages from the J774A mouse cell line.1, and we found that they are susceptible to infection by the two *Bacillus* species. However, both species were eliminated as the infection progressed. Using confocal microscopy, we identified the formation of METs from the first hours of infection, which were characterized by the presence of myeloperoxidase (MPO) and citrullinated histone (Hit3Cit). Quantitative data on extracellular DNA release were also obtained; release was observed starting in the first hour of infection. The induction of METs by *B. licheniformis* caused a significant decrease in the colony-forming units (CFU) of *Staphylococcus aureus*. The induction of METS by bacteria of the *Bacillus* genus is a mechanism that participates in controlling the probiotic and potentially pathogenic bacteria such as *S. aureus*. The induction of METs to control pathogens may be a novel mechanism that could explain the beneficial effects of probiotics of the genus *Bacillus.*

## 1. Introduction

Probiotics are live microorganisms that are administered in adequate amounts to a host in order to conf er health effects [1]. These are not considered pathogens for humans. The use of probiotics has spread in animals and humans, where they have been used as auxiliary treatments in infections, especially gastrointestinal [2]. The most used genera include *Lactobacillus* and *Bifidobacterium*, although some species of the genus *Bacillus* have also been considered [3,4].

*Bacillus* bacteria are saprophytes. They are found in various environmental conditions, as well as in water and soil. The growth conditions are varied within the genus, but they are capable of producing spores [4]. Most *Bacillus* species are not considered risky for humans, so they are used for the fermentation of some foods. However, species such as *B. cereus* and *B. anthracis* are considered as food contaminants or as infectious agents, restricting their use as probiotics [4,5,6].

*B. subtilis* and *B. licheniformis* belong to the group of *B. subtilis;* these species are considered to be, and used as, probiotics due to their ability to produce enzymes and secondary metabolites that have been known to inhibit the growth of pathogenic microorganisms [7,8]. They can maintain a symbiotic relationship with plants and are used for the fermentation of food. They are also widely used as probiotics in the veterinary area [9,10,11,12]. However, reports of infections by *Bacillus* species have created mistrust regarding their use as probiotics due to the production of toxins associated with diarrheal disorders and the resistance to antibiotics that they may present [13]. In 2008, Sorokulova [14] and collaborators evaluated the safety of two *Bacillus* species (*B. subtilis* and *B. licheniformis*) used in a commercial probiotic, finding that none of them contained enterotoxin and hemolysin genes (Nhe, Hbl, enterotoxin T, cytotoxin K) unlike *B. cereus*. Because the genus *Bacillus* is not considered part of the intestinal microbiota, the aforementioned authors evaluated the adhesion capacity of said strains, resulting in the inability to generate an infection through invasiveness. It was also observed that there was no damage to the organs of mice, rabbits, or pigs that were administered with large amounts of *Bacillus*. It should be noted that these bacilli were eliminated two days after administration, although the details of the mechanism of elimination of bacteria by the host have not been described [14].

The experimental infection of *B. licheniformis* has been reported in the mouse model, where the mice with an intact immune system presented only mild lesions on the days of observation, with greater damage occurring in animals with compromised immune systems. In this model, the presence of the bacteria was observed in lung and liver macrophages [15]. To date, infection by *B. subtilis* in macrophages has not been described. It was recently shown [16] that *Bacillus* capsules are capable of activating the immune response through Toll-like receptors (TLRs) and, in this way, promote the activation of monocytes and dendritic cells. In addition, the immune stimulatory activity of probiotic *B. subtilis* has been demonstrated in the elderly [17].

Macrophages are cells that belong to the innate immune system. They are capable of engulfing and using mechanisms that allow the elimination of infectious agents, such as bacteria. By engulfing microorganisms, macrophages activate machinery that allows the production of metabolites such as nitric oxide (NO) and reactive oxygen species (ROS), which contribute to the degradation of the pathogen. Extracellular traps (ET) are considered to be network-type structures made up of DNA, and their production has been related to the presence of ROS. These traps can be formed by neutrophils and macrophages in response to infection with bacteria, fungi, and parasites, where the main function is to prevent the spread of microorganisms [18,19,20,21,22,23].

Therefore, the objective of this work was to establish whether *B. licheniformis* and *B. subtilis* are capable of promoting the formation of macrophage extracellular traps (METs), and to analyze whether their induction can control the growth of microorganisms considered pathogens such as *Staphylococcus aureus*.

## 2. Materials and Methods

### 2.1. J774A.1 Macrophage Culture

Murine macrophages of the J774A.1 cell line were used (ATCC TIB-67), kept in RPMI medium with L-Glutamine (Sigma-Aldrich, St. Louis, MI, USA) with 7% Fetal Bovine Serum (SFB) (BY-PRODUCTOS, Guadalajara, México) and the mixture of the antibiotics Penicillin-Streptomycin (Biowest, Riverside, MI, USA). The cells were incubated at 37 °C with an atmosphere of 5% CO_2_. For the infection experiments, the cells were used with a medium without antibiotics and with 2% FBS.

### 2.2. Cultivation of Bacillus licheniformis and Bacillus subtilis

Two species of the *Bacillus* genus were used, *B. licheniformis* and *B. subtilis*, which were part of the bacterial biocollection of the Unidad Xochimilco Metropolitan Autonomous University (UAM-X). The identity of the species was corroborated by mass spectrometry with VITEK-MS equipment (bioMeriux, Marcy l’Etoile, France ). Prior to the infection experiments, the bacteria were grown in brain heart infusion (BHI) broth (BD, Franklin Lakes, NJ, USA) for 12 h at 37 °C and adjusted to tube 1 of the McFarland Nephelometer. To verify the purity of the bacteria, the cultures were seeded on trypticase soy agar (TSA, BD, México City, México).

### 2.3. Obtaining Intracellular CFU and Cell Viability of Infected Macrophages

Macrophage infection with *B. licheniformis* and *B. subtilis* was performed with a multiplicity of infection (MOI) of 10:1 (bacteria:cells), using 250,000 cells per well, in 24-well plates. The bacteria were incubated with the cells for 2 h at 37 °C in an atmosphere of 5% CO_2_. Next, the extracellular bacteria were eliminated with Amikacin [80 µg/mL] for 2 h, and then a maintenance medium was used with an amikacin concentration [15 µg/mL]. Samples were taken 2, 6, 8, 12, and 24 h post-infection. To recover the intracellular bacteria, the cells were lysed during 15 min with 0.5 mL of sterile distilled water. Later, 0.5 mL of a sterile bovine serum albumin (Sigma-Aldrich) solution at 4% were added, recovering the lysate and freezing it at −70 °C until the determination of CFU. Once every sample was collected, the lysates were thawed, and serial dilutions (-1, -2, and -3) were made; a 10 μL drop of each dilution was seeded in TSA medium and incubated at 37 °C for 12 h. 

To determine macrophage viability, monolayers were prepared and infected as described above. To distinguish dead cells from living cells, trypan blue exclusion staining was performed. Viability was determined at 2, 4, 6, 8, 12, and 24 h post-infection.

### 2.4. Analysis of Cellular Alterations and Observation of the Formation of Extracellular Traps

Alterations in the cell morphology of infected macrophages were evaluated in short infection times (1, 2, and 3 h). For this evaluation, macrophage monolayers were prepared as described above, but prior to the preparation of the monolayers, sterile coverslips were placed in each well of the plate. At the end of each analysis time, the coverslips were removed and fixed with 4% paraformaldehyde (PFA), then gently washed with sterile Hanks’ solution. Subsequently, 0.1 mMol of Sytox Green was added, and the preparations were mounted with VECTASHIELD (Vector Laboratories, Burlingame, CA, USA). The preparations were observed in a confocal microscope (LSM5 Pascal, Zeiss, Oberkochen, Germany). In all the experiments, zymosan treatment was included as a positive control for the induction of extracellular traps in macrophages [19], for which monolayers were prepared as indicated above. Prior to treatment, the cells were carefully washed 2 times with Hanks’ saline without phenol red. Subsequently, to each well that would receive the treatment, the suspension of zymosan (Sigma-Aldrich) was added at a concentration of 1 mg, resuspended in 1 mL of sterile phosphate buffer solution (PBS), incubated for 1, 2, and 3 h. 

Detection of the proteins histone 3-citrullinated (Hit3Cit) and myeloperoxidase (MPO) was also analyzed at times of 1, 2, and 3 h post-infection. Cells were prepared on coverslips, as noted above. Once the infection time had concluded, the cells were fixed with PFA and subsequently permeabilized with 0.25% sodium dodecyl sulfate (SDS) (BioRad, Hercules, CA, USA). In the case of Hit3Cit, the mouse anti-Hit3Cit antibody (Abcam, Cambridge, UK) was added, it was diluted 1:150 in 4% Albumin and then incubated for 24 h under refrigeration. Subsequently, a secondary antibody coupled to fluorescein isothiocyanate (FITC) (anti-rabbit-FITC 1:100, KPL) was used, incubating for 2 h at room temperature in the dark. In the case of MPO, an anti-MPO antibody coupled to phycoerythrin (PE) (1:150, Santa Cruz, Dallas, TX, USA) was used, which was incubated at room temperature for two hours under dark conditions. Finally, the preparations were mounted with VECTASHIELD with DAPI (4′,6-diamino-2-phenylindole) (Vector). The preparations were observed with a confocal microscope. 

### 2.5. Determination of ROS

The p-nitro blue tetrazolium reduction technique (NBT, Sigma-Aldrich) was used to evaluate ROS production, following the protocol described by García et al., 2011 [24]. After infecting the cells, 0.1% NBT was placed in each well and incubated for 30 min at 37 °C in an atmosphere of 5% CO_2_. At the end of the time, the NBT was removed, followed by washing with Hanks’ solution, to later add a 500 µL of a V/V mixture of potassium hydroxide (2 M KOH) and dimethyl sulfoxide (DMSO). The absorbance reading was performed using a microplate reader (Multiskan MS, LabSystems, Thermo Fisher Scientific, Waltham, MA, USA) at a wavelength of 600 nm. 

### 2.6. Quantification of Extracellular DNA

The quantification of the extracellular DNA was performed after the cells were infected with the *Bacillus* species, following the protocol of Campillo et al., 2016 [25]. Briefly, after each study time, the culture medium was gently removed from each well, subsequently placing 1 U of DNAse and incubating for 30 min at 37 °C in an atmosphere of 5% CO_2_. Next, 400 μL of RPMI medium without phenol red was added, continuing with the staining with 0.1 µMol of Sytox Green, and incubating for an additional 5 min. Finally, 200 μL were taken from each well, and the relative fluorescence units (RFU) were determined in a Fluorometer (LabSystems, Thermo Fisher Scientific) at an excitation wavelength of 485 nm and an emission of 538 nm.

### 2.7. Inhibitory Activity of Bacillus-Induced METs against Staphylococcus aureus

To establish the inhibitory effect of *Bacillus*-induced METs on potential human pathogens, a challenge test was performed with *Staphylococcus aureus*, as described by Campillo et al., 2016 [25]. In brief, a strain of *Staphylococcus aureus* (ATCC 25923) was used. The bacterium was cultured in Mueller Hinton broth (Sigma-Aldrich) for 24 h at 37 °C. Initially, the formation of METs was induced with the two species of *Bacillus,* infecting the macrophages in 24-well microplates as described above. After one hour of infection, very gentle washes were carried out with Hanks’ saline solution, later adding the suspension of *S. aureus* at an MOI of 5:1, allowing an incubation of 30, 60, and 90 min at 37 °C, with 5% CO_2_. At the end of the infection time, the supernatant was collected, and serial dilutions (-1, -2, and -3) were performed, seeding them on trypticase soy agar (TSA, BD), incubating the plates for 24 h at 37 °C, and then counting the observed colony forming units (CFU).

### 2.8. Statistical Analyses 

The studies related to ROS production, quantification of DNA release, and effect of the METs on survival of *S. aureus*, were performed in triplicate. All data are presented as mean ± standard error of the mean (SEM). Data were analyzed with GraphPad Prism v. 5.1 (GraphPad Software, Inc., San Diego, CA, USA) using a repeated-measures ANOVA test followed by a *t*-test as post hoc with the Bonferroni correction. *p* < 0.05 and *p* < 0.001 were considered significant for statistical tests. 

## 3. Results

### 3.1. B. licheniformis and B. subtilis Are Eliminated by Macrophages, and They Induce the Formation of METs

**Figure 1:**
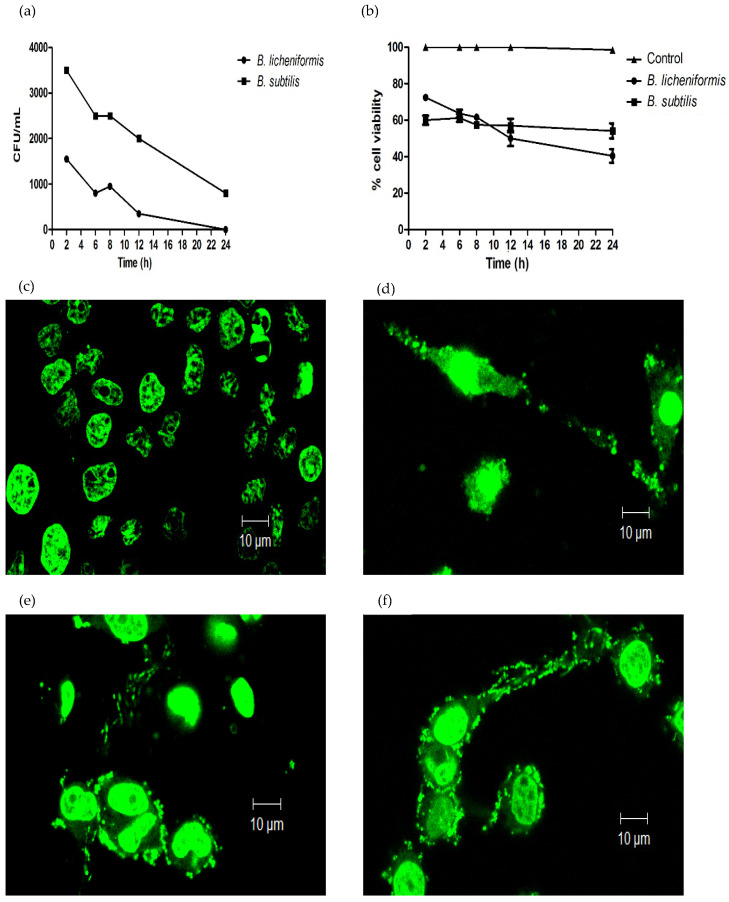
Macrophages of the J774A.1 cell line. When infected with *Bacillus licheniformis* and *Bacillus subtilis*, they die as a result of infection and produce METs. (**a**) Intracellular *B. licheniformis* and *B. subtilis* colony-forming units were determined at different infection times. (**b**) Macrophage cell viability was determined by the trypan blue exclusion assay at different infection times. As a consequence of the infection, there were nuclear and cellular changes, which were evidenced by Sytox Green staining after 3 h of infection and observed by confocal microscopy (**c**) Control cells. (**d**) Zymosan treated cells. (**e**) *B. licheniformis* and (**f**) *B. subtilis.* 630× magnification. Representative results of three experiments.

*B. licheniformis* and *B. subtilis* are probiotics, which improve the health of the host, promoting the restoration of the microbiota and strengthening the immune response. These bacteria have been described as non-pathogenic [3], which is why we investigated whether they had the ability to infect macrophages. We used mouse macrophages of the J774A.1 cell line as a study model and infected them with both bacteria. We observed that macrophages were susceptible to infection with both species, obtaining intracellular CFUs after 2 h. However, the internalized bacteria were gradually eliminated by macrophages, achieving a complete elimination of *B. licheniformis* after 24 h of infection and an elimination of more than 80% of *B. subtilis* in the same timeframe (Figure 1a). Next, we evaluated the viability of the macrophages during the time of infection. It was observed that there was a decrease in cell viability throughout the infection, and after 24 h, around 50% of the cells died from both infections (Figure 1b).

Due to the loss of cellular viability of the macrophages, morphological changes of the cells were observed in the first hours of infection. When analyzing infected cells under the light microscope, multiple cell changes suggestive of cell death were observed. In order to appreciate possible nuclear alterations and the possible presence of cytoplasmic DNA, the samples were stained with Sytox Green (SG), a dye that is intercalated between nucleic acids, generating a green fluorescence (Figure 1c–f). The nuclei of uninfected macrophages were round or oval and of a homogeneous size. No dispersed nuclear material was observed in the cytoplasm of the cells (Figure 1c). Zymosan is one of the previously reported inducers of METs [19], which is why it was used as a positive control for the induction of METs in our experimental model. We observed that the macrophages treated with zymosan presented alterations in nuclear morphology, evidenced by a more homogeneous staining with SG. Interestingly, a fluorescent mark was observed at the cytoplasmic level and in the cell prolongations formed during infection. These images were compatible with the formation of METs (Figure 1d). Similar to what was observed with zymosan, macrophages infected with *Bacillus* species presented cellular alterations, with the presence of more compact nuclear material and the presence of genetic material in the cytoplasmic space and in cell prolongations (Figure 1e,f).

**Figure 2:**
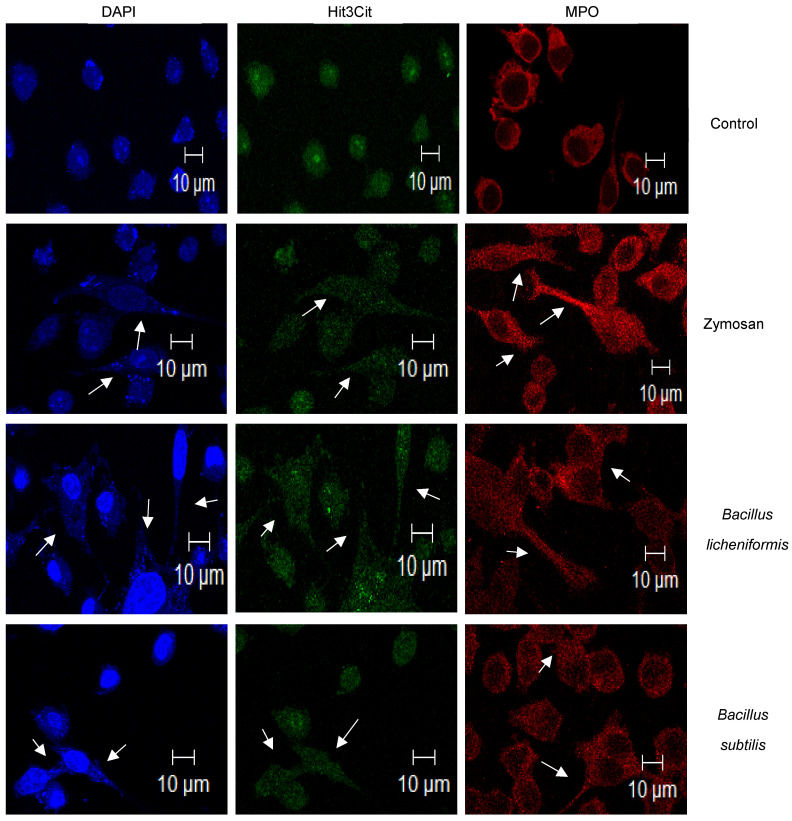
*Bacillus licheniformis* and *Bacillus subtilis* induce the formation of macrophage extracellular traps. Macrophages were infected or treated with zymosan for 3 h. Immunofluorescence showed the presence of nuclear and cellular distribution of Hit3Cit (green), MPO (red), and nuclear DNA (blue), fundamental components of METs. Arrows pointed to METs extensions containing DNA, Hit3Cit, and MPO. Representative images of three experiments. The preparations were observed under a confocal microscope at 630× magnification.

Based on observations with SG staining, which suggested that METs were induced due to infection, we proceeded to search for two proteins that have been described to be located in extracellular traps: Hit3Cit and MPO [26]. The MET-induction process involves the citrullination of histones, thus preventing histone from maintaining compacted chromatin and thereby presenting nuclear material at the cytoplasmic level and in network-type extracellular structures [27,28]. Myeloperoxidase (MPO) is an enzyme normally found in the granules of neutrophils and macrophages, and it has been found to be present in extracellular traps during the MET formation process [29]. In order to establish the simultaneous presence of DNA, Hit3Cit, and MPO by confocal microscopy, a triple stain was performed on those cells which were uninfected, those treated with zymosan, and those infected with both species of *Bacillus*. As can be seen in Figure 2, in the control cells stained with SG and Hit3Cit, both marks were observed concentrated in the cell nucleus (blue and green staining), while the staining that evidenced MPO (red) was found at the perinuclear level (Figure 2). In cells stimulated with zymosan and those infected with both bacteria, cellular changes were corroborated with the presence of nuclear material at the cytoplasmic level and in cell prolongations (blue staining). In the same way, the Hit3Cit and MPO mark presented a change in its cellular distribution. Hit3Cit was observed to be distributed throughout the cytoplasm (green mark) and in cell prolongations. Similarly, MPO (red mark) was distributed throughout the cell space and in the cell prolongations formed (Figure 2). The morphological changes and the observed cellular distribution of these proteins and DNA confirm that the formation of METs was induced during infection with probiotics.

### 3.2. ROS-Production during Infection and Induction of METs

**Figure 3:**
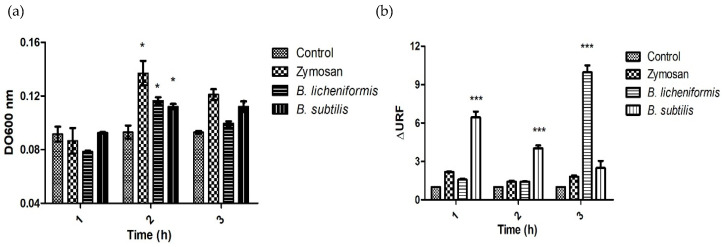
Macrophages infected with probiotics produce ROS and METs. (**a**) ROS production by macrophages infected with *Bacillus* species and treated with zymosan. (**b**) Quantification of DNA released by macrophages infected with *Bacillus* species and treated with zymosan determined by SYTOX fluorometry. Representative results of three experiments. ANOVA test for repeated measures was performed, followed by a post hoc *t*-test with a Bonferroni correction. * *p* < 0.05, *** *p* < 0.001.

Reactive oxygen species have been related to the production of extracellular traps of neutrophils and macrophages [30,31]. Therefore, we evaluated whether the infected cells produced ROS. To do so, we performed the NBT reduction test in which we observed that the control cells had a basal ROS production at the three study times. In comparison, the macrophages treated with zymosan presented a high production of ROS with respect to the unstimulated cells at 2 h and 3 h post-treatment. In the case of cells infected with *Bacillus*, it was observed that the highest production of ROS was at 2 h for both species (Figure 3a). As DNA is released as a result of METs formation, it can be quantified by fluorometry. The presence of extracellular DNA was determined and was found only in the case of infections. With *B. subtilis,* extracellular DNA was found at 1 h and 2 h post-infection, while *B. licheniformis* induced the highest production of extracellular DNA, although it was only towards the third hour of observation (Figure 3b).

### 3.3. METs Induced by B. licheniformis Inhibit the Growth of Staphylococcus aureus

**Figure 4:**
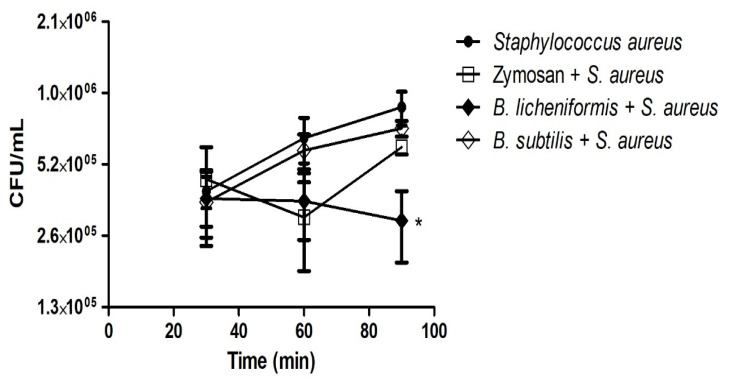
*B. licheniformis* reduces the CFU/mL of *Staphylococcus aureus.* The formation of METs was induced in macrophages infected with *Bacillus* species for 1 h. The METs formed were challenged with *S. aureus* for 30, 60, and 90 min. The CFU/mL of *S. aureus* were determined. The repeated measures ANOVA test was performed, followed by a post hoc *t*-test with a Bonferroni correction. * *p* < 0.05.

*B. licheniformis* and *B. subtilis* are probiotics that have been used to prevent the growth of microorganisms considered pathogens such as *E. coli* or *Salmonella* [32,33]. The results obtained so far indicate that both probiotics induce the production of METs. This mechanism may be responsible for or contribute to the efficient elimination of *Bacillus* by macrophages (Figure 1a). However, we wonder if the METs induced by *Bacillus* have the property of killing other microorganisms, including pathogenic bacteria such as *S. aureus*, a Gram-positive bacteria that causes various types of infections in the respiratory tract, wounds, skin, and enterocolitis post-antibiotic therapy [34,35]. To corroborate this hypothesis, the macrophages were first infected with *Bacillus* species and with zymosan, then incubated for 1 h, thus allowing the formation of METs. Next, the medium was removed, and the suspension of *S. aureus* was then added. The CFUs were determined at 30, 60, and 90 min after contact with the METs. The recovery of *S. aureus* that was in contact with macrophages without stimulation showed an increase in CFUs at times 60 and 90 min after contact. Similarly, we observed the recovery of *S. aureus* in contact with METs induced by *B. subtilis*, although its survival tendency was lower than with macrophages without stimulation. The METs induced with zymosan showed an inhibitory effect on the recovery of CFU from *S. aureus* at 60 min. However, at 90 min, there was an important recovery in the growth of *S. aureus*. Finally, the METs induced by *B. licheniformis* inhibited the growth of *S. aureus* in an important, constant, and statistically significant way (Figure 4).

## 4. Discussion

The use of probiotics at present has allowed them to be considered an adjuvant treatment in various types of intestinal and inflammatory disorders, including diarrhea, since they participate in the restoration and conservation of the microbiota, which actively participates in the modulation of the immunological system [3,36,37,38]. The pharmaceutical and commercial presentations of bacterial probiotics include vegetative bacterial and sporulated forms. The latter offer advantages since their high resistance allow spores to germinate and vegetative bacteria to be generated in the intestine, facilitating their establishment and eventually exerting their beneficial effect. However, not only vegetative forms have beneficial effects. It has been described that *B. subtilis* spores are activators of the immune response [16]. In this study, we worked with the vegetative form of *B. licheniformis* and *B. subtilis*, bacteria that are considered to be non-pathogenic. We, therefore, decided to establish their ability to infect macrophages. It was found that the macrophages internalized both bacteria. After the infection time elapsed, the macrophages promoted their elimination. Specifically, the macrophages completely eliminated the bacterial growth of *B. licheniformis* (Figure 1a). However, infection with both bacteria had a significant effect on macrophage viability. At the time of infection analyzed, about 50% of macrophages died as a result of the infection. To date, there are no previous studies that describe a similar effect on the interaction of macrophages with both species of *Bacillus.* Currently, various forms of cell death have been described, including programmed and non-programmed cell death. One form of non-programmed death is ETosis, cell death that occurs mainly in cells of the immune system such as neutrophils or macrophages and occurs as a result of the recognition, internalization, or response to pathogens by neutrophils or macrophages among other cell lines. Important cellular and nuclear changes occur in this type of cell death, including the citrullination of histones, chromatin decondensation, the rupture of nuclear and cell membranes, the formation of network-type extracellular protrusions, which, in addition to containing DNA, contain antimicrobial proteins derived from cell granules. These structures are called extracellular traps and have been reported to have antimicrobial activity [18,39]. When analyzing the viability of infected macrophages, the observed morphological changes, suggested that the cells could be dying from ETosis and consequently forming extracellular traps. Zymosan was used as a positive control for the induction of METs, a stimulus that has previously been shown to have this property [19]. SG staining revealed the cellular and chromatin changes resulting from infection with *B. licheniformis* and *B. subtilis*, mainly observing nuclear changes, the presence of cytoplasmic DNA, and the formation of cell protrusions with the presence of DNA (Figure 1e,f). These changes were also observed in macrophages treated with zymosan (Figure 1c). To characterize the METs induced by infection with probiotics, we searched for the presence of Hit3Cit and MPO in the infected cells. There was an apparent change in the cytoplasmic and/or nuclear distribution of these proteins. Both proteins were dispersed in the cellular cytoplasm and in the cellular protrusions observed. A similar distribution was observed in macrophages treated with zymosan (Figure 2). In this way, the results confirm that probiotics induce the formation of METs after an infection.

Macrophages are essential cells in the innate immune response. In response to infection, they engulf bacteria and activate various mechanisms to control pathogens. These mechanisms include the production of ROS [40]. In the case of macrophage infection by the probiotics under study, a significant production of ROS by macrophages was observed with both bacteria (Figure 3a). In addition to antimicrobial effects, ROS production resulting from macrophage activation as a response to infection has also been responsible for the induction of extracellular trap formation [21,39,40]. Therefore, considering the results obtained, it is feasible to propose that the ROS produced by macrophages in response to infection contributed to the elimination of *Bacillus* species and also contributed to the formation of METs and the death of macrophages. The highest ROS values, specifically those statistically significant at 2 h, were those induced by both species. Interestingly, *B. liqueniformis* was eliminated more efficiently by macrophages compared to *B. subtilis* (Figure 1a and Figure 3a). Similarly, *B. licheniformis* was the species that induced the highest DNA release, specifically at 3 h post-infection. This could indicate that *B. licheniformis* is a better METs-inducer species, although this event was counterproductive for the bacteria since it more efficiently promoted bacterial elimination.

The induction of METs-formation by probiotics has not been reported to date. This work is the first description of this phenomenon. Vong et al. reported in 2014 [41] that the probiotic *Lactobacillus rhamnosus* GG, an important producer of antioxidants, does not have the property of inducing the formation of neutrophil extracellular traps (NETs). On the contrary, the bacteria presented an inhibitory effect on the formation of NETs induced in neutrophils with phorbol myristate acetate (PMA) or by infection with an infectious agent like *S. aureus*. In the study by Vong et al. (2014), the inhibition of NETs was attributed to the property of the probiotic to decrease ROS levels due to its production of antioxidants and thus prevent the formation of NETs. In this manner, the modulation of the formation of NETs or METs by probiotics is a fundamental element in exerting their beneficial biotherapeutic effects.

The *Bacillus* genus, unlike the *Lactobacillus* genus, is not part of the human microbiota, which highlights the importance of demonstrating that it is safe as a probiotic. There is evidence that *B. licheniformis* and *B. subtilis* do not produce non-hemolytic enterotoxins, hemolysins, or cytotoxins, nor do they generate invasive infections [14,42]. Our results contribute to establishing the safe use of both species since, despite the fact that they can infect macrophages, this infection is eventually resolved by the cells. In this manner, the macrophages help control and prevent the systemic dissemination of probiotics, containing their localization at the intestinal level.

The use of spores or products fermented by *B. licheniformis* and/or *B. subtilis* has resulted in a benefit to the consumer. For example, they have been used in the poultry industry to promote the growth of chickens and to eliminate pathogenic infectious agents such as *Salmonella* [9,32]. In another more recent study, it was described that *Bacillus* could inhibit the growth and adhesion of enterotoxigenic *E. coli* (ETEC) [33]. Considering the possible antagonistic effect of probiotics of the *Bacillus* genus against bacterial pathogens and that this could be due to the antimicrobial effects described for METs [25], we carried out an in vitro challenge of METs induced by *Bacillus* against *S. aureus*, resulting in a decrease in CFU/mL, which was significant in the case of METs formed from infection with *B. licheniformis* (Figure 4). In the above results, the species *B. licheniformis* was the probiotic that induced a high production of ROS and the highest release of DNA. These conditions could be optimal for achieving the formation of METs with antimicrobial activity.

## 5. Conclusions

In conclusion, the probiotics of the genus *Bacillus*, such as *B. subtilis* and *B. licheniformis*, are safe bacteria that, if disseminated, could be taken up by macrophages and be efficiently eliminated. Their beneficial effects on health are provided in their sporulated form. They are also provided through the production of metabolites and, in their vegetative form, inducing the production of METs that have inhibitory effects against pathogenic bacteria.

## Data Availability

The data presented in this study are available on request.
